# Complementary and alternative medicine (CAM) supplements in cancer outpatients: analyses of usage and of interaction risks with cancer treatment

**DOI:** 10.1007/s00432-021-03675-7

**Published:** 2021-07-06

**Authors:** Clemens P. J. G. Wolf, Tobias Rachow, Thomas Ernst, Andreas Hochhaus, Bijan Zomorodbakhsch, Susan Foller, Matthias Rengsberger, Michael Hartmann, Jutta Huebner

**Affiliations:** 1 Klinik für Innere Medizin II, Hämatologie und Internistische Onkologie, Universitätsklinikum Jena, Am Klinikum 1, 07747 Jena, Germany; 2 Klinik für Innere Medizin II, Hämatologie und Internistische Onkologie, Pneumologie, Universitätsklinikum Jena, Am Klinikum 1, 07747 Jena, Germany; 3 Klinik für Innere Medizin II, Hämatologie und Internistische Onkologie, Konservative Tagesklinik des UniversitätsTumorCentrums (UTC), Universitätsklinikum Jena, Am Klinikum 1, 07747 Jena, Germany; 4 Onkologische Kooperation Harz, Kösliner Straße 14, 38642 Goslar, Germany; 5 Klinik für Urologie, Universitätsklinikum Jena, Am Klinikum 1, 07747 Jena, Germany; 6 Klinik und Poliklinik für Frauenheilkunde und Fortpflanzungsmedizin, Universitätsklinikum Jena, Am Klinikum 1, 07747 Jena, Germany; 7 Apotheke des Universitätsklinikums, Universitätsklinikum Jena, Am Klinikum 1, 07747 Jena, Germany; 8 Klinik für Innere Medizin II, Hämatologie und Internistische Onkologie, Integrative Onkologie, Universitätsklinikum Jena, Am Klinikum 1, 07747 Jena, Germany

**Keywords:** Drug interactions, Complementary and alternative medicine, Cancer treatment, Chemotherapy, Cancer outpatients

## Abstract

**Purpose:**

The aim of our study was to analyze the use of complementary and alternative medicine (CAM) supplements, identify possible predictors, and analyze and compile potential interactions of CAM supplements with conventional cancer therapy.

**Methods:**

We included outpatient cancer patients treated at a German university hospital in March or April 2020. Information was obtained from questionnaires and patient records. CAM–drug interactions were identified based on literature research for each active ingredient of the supplements consumed by the patients.

**Results:**

37.4% of a total of 115 patients consumed CAM supplements. Potential interactions with conventional cancer treatment were identified in 51.2% of these patients. All types of CAM supplements were revealed to be a potential source for interactions: vitamins, minerals, food and plant extracts, and other processed CAM substances. Younger age (< 62 years) (*p* = 0.020, φc = 0.229) and duration of individual cancer history of more than 1 year (*p* = 0.006, φc = 0.264) were associated with increased likelihood of CAM supplement use. A wide range of different CAM supplement interactions were reviewed: effects of antioxidants, cytochrome (CYP) interactions, and specific agonistic or antagonistic effects with cancer treatment.

**Conclusion:**

The interaction risks of conventional cancer therapy with over-the-counter CAM supplements seem to be underestimated. Supplements without medical indication, as well as overdoses, should be avoided, especially in cancer patients. To increase patient safety, physicians should address the risks of interactions in physician–patient communication, document the use of CAM supplements in patient records, and check for interactions.

## Introduction

Complementary and alternative medicine (CAM) comprises all modalities that are used instead of (alternative medicine) or in addition to (complementary medicine) conventional and more stringently evidence-based medicine by patients (NCCIH 2018; Teichfischer and Muenstedt 2011). These include, for example, the use of herbal substances, non-prescribed use of supplements such as vitamins or minerals, homeopathy, Chinese medicine, massage, acupuncture, prayer, and more.

The use of CAM is widespread among cancer patients. Exact frequencies vary between different studies, e.g., 29% Firkins et al. (2018), 34% Wode et al. (2019), 36% Molassiotis et al. (2005), 40% Horneber et al. (2012), 49% Berretta et al. (2017), 59% Micke et al. (2009). Younger age, female gender, and higher educational level (Micke et al. 2009; Molassiotis et al. 2005; Wode et al. 2019), as well as breast cancer diagnosis (Micke et al. 2009), are discussed as predictive parameters for CAM use. Since the 1970s, the number of patients using CAM has been increasing (Horneber et al. 2012).

Consuming supplements including vitamins, minerals, and plant extracts is the most commonly used CAM modality in Europe and the United States (Alsanad et al. 2016; McCune et al. 2004; Micke et al. 2009; Molassiotis et al. 2005; Naing et al. 2011), which can potentially interact with conventional cancer therapy. A high prevalence of potential CAM–drug interactions with conventional anticancer drugs has been reported in several studies, but numbers are heterogeneous: e.g., 55% Firkins et al. (2018), 65% Zeller et al. (2013), 85% Loquai et al. (2017).

The aim of our study was to analyze the use of CAM supplements by calculating associations with demographic data and to identify possible predictors, as well as to analyze and compile potential interactions of CAM substances with conventional cancer therapy in terms of their probabilities.

Critical issues with CAM and its potential interactions are the lack of in vivo data and clinical studies investigating the clinical relevance of CAM substance interactions. The potential for substance interactions often can only be estimated from in vitro experiments or murine models (Huebner 2012).

In fact, most if not all patients want to avoid harming themselves when using complementary medicine. In a study of outpatient cancer patients by McCune et al. (2004), more than 85% conceded that they would stop using a CAM drug or ask their physician for advice if interactions were known. However, potential interactions are still common, presumably reflecting patients’ and physicians’ unawareness regarding the risks of natural substances, most of which are over-the-counter drugs distributed not only by pharmacies but also via the internet.

## Methods

### Patients

Randomly selected cancer patients with different cancer diagnoses were included in our cross-sectional study. All patients were treated for their cancer as outpatients at Jena University Hospital, Germany in March or April 2020. They were informed and agreed to participation, data processing, and publication of results.

### Data collection

A standardized questionnaire was used to collect information on demographic data, type of cancer diagnosis and time of initial diagnosis, use of additional substances, current medication, and way of medication intake. Non-prescribed supplements consumed by patients for health purposes were considered as CAM supplements. If vitamins were consumed but not specified, intake of a vitamin blend containing the vitamins listed in Table 4 was assumed. Other CAM modalities such as prayer, acupuncture, or massages were not included. Additional information on cancer diagnosis, current cancer therapy, and concomitant medication was obtained from patient records.

### Evaluation of interactions

For each CAM substance used by the patients, the literature was searched for possible interactions of the individual active ingredients of the respective CAM supplement with conventional, physician-prescribed cancer treatment drugs, including chemotherapy, hormone therapy, and immunotherapy (target therapy). The likelihood of interactions found regarding CAM supplements was ranked from unlikely (0) to possible (1) to likely (2). In the case where the likelihood of a particular interaction was heterogeneously assessed in the literature, when the respective sources were considered to be methodologically correct, two of the authors (Wolf and Huebner) discussed the arguments and decided on a classification considering the conditions of the respective studies and the reasons given by the authors. Consensus was reached in each case.

### Statistics

Data were compiled using Microsoft Office Excel 2016 and statistically analyzed using IBM SPSS Statistics 27. Associations were analyzed mainly by calculating correlations. For correlations of categorical and ordinal variables, Cramér’s V (φc) was used after performing the Fisher’s Exact Test or Fisher-Freeman-Halton Test, and for correlations of metric and categorical or ordinal variables, Eta Squared (*η*^2^) and a significance test by performing an analysis of variance was used. Demographic variables that were metric variables, such as age or number of drugs consumed, were recoded into dichotomous variables, as shown in Table 3 because significance testing of such correlations with an analysis of variance may only be performed if the dependent variable is the metric variable. Binary logistic regression was calculated for potential parameters predicting CAM use and for the potential of CAM-drug interactions involving conventional anticancer medication.

In a second article﻿, we evaluate and report patient data regarding the frequency and probability of interactions. We consider on a variety of causes of interactions, such as anticancer drugs, supportive medication, drugs prescribed for comorbidities' treatment, and nutrition (Wolf et al. 2021).

## Results

One hundred fifteen patients participated in our study. Demographic data are presented in Table 1. The mean age was 61 years (SD = 13.3). 40.9% of patients were male. The most common diagnosis categories (Table 2) were breast cancer (*n* = 25), other gynecological cancers (*n* = 15) such as ovarian cancer, cervical cancer, or endometrial cancer, multiple myeloma (*n* = 15), and leukemia (*n* = 10).

**Table 1:** Demographic data (*n* = 115)

Age	
Median (Range)	63 (18–86) years
Patients older than 61 years, *n*	65 (56.5%)
Gender, *n*	
Male	47 (40.9%)
Female	68 (59.1%)
Marital status, *n*	
Single	11 (9.6%)
Firm relationship	8 (7.0%)
Married	76 (66.1%)
Divorced	6 (5.2%)
Widowed	13 (11.3%)
No data	1 (0.9%)
Children, *n*	
Median (Range)	1 (0–4)
Patients with 1 or more children	86 (74.8%)
Minor children, *n*	
Median (Range)	0 (0–2)
Patients with 1 or more minor children	13 (11.3%)
School leaving qualification, *n*	
No degree	1 (0.9%)
After 8th grade (Hauptschulabschluss)	10 (8.7%)
After 10th grade (Mittlere Reife)	41 (35.7%)
After 12th or 13th grade (Abitur)	31 (27.0%)
No data	32 (27.8%)
Time since initial cancer diagnosis	
Median (Range)	17 months (< 1 month—26 years)
Patients with time > 1 year, *n*	69 (60.0%)
Drugs prescribed by physicians, *n*	
*n*	1191
Median (Range)	10 (1–23)
Patients with 10 or more drugs prescribed	68 (59.1%)
Drugs prescribed for cancer treatment, *n*	
*n*	279
Median (Range)	2 (0–5)
Amount of CAM compounds consumed, *n*	
*n*	117
Median (Range)	0 (0–12)
Types of CAM supplements consumed, *n*	
Patients using vitamin supplements	22 (19.1%)
Patients using minerals	24 (20.9%)
Patients using certain food	13 (11.3%)
Patients using other processed CAM substances	19 (16.5%)

**Table 2:** Frequency of diagnosis categories and distribution of CAM supplement use

Cancer diagnosis	Patients	CAM users, *n* (%)	Number of compounds consumed in the group of CAM using patients, n (average per user)
Breast cancer	25 (21.7%)	9 (36.0%)	30 (3.3)
Other gynecological cancer	15 (13.0%)	6 (40.0%)	12 (2.0)
Multiple myeloma	15 (13.0%)	8 (53.3%)	30 (3.8)
Leukemia	10 (8.7%)	0 (0%)	0 (0)
Pancreatic cancer	8 (7.0%)	4 (50.0%)	7 (1.8)
Gastrointestinal cancer	8 (7.0%)	3 (37.5%)	11 (3.7)
Renal cancer	8 (7.0%)	1 (12.5%)	4 (4)
Cholangiocellular carcinoma	6 (5.2%)	3 (50.0%)	5 (1.6)
Lung cancer	6 (5.2%)	2 (33.3%)	3 (1.5)
Malignant lymphoma	5 (4.3%)	2 (40.0%)	5 (2.5)
Others	9 (7.8%)	5 (55.6%)	10 (2)
*n*	115	43 (37.4%)	117 (2.7)

Non-prescribed supplements, which were considered CAM supplements, were taken by 43 patients (37.4%). These patients took a total of 117 CAM compounds. Twenty-two patients consumed vitamin supplements, 24 supplemented minerals including trace elements, 19 used certain food or plant extracts like Brazil nuts, Chinese herbs, ginger, medicinal mushrooms, mistletoe, teas, turmeric, spirulina, or others, and 9 patients consumed other processed CAM compounds like homeopathy or probiotics. Seven of the 43 CAM supplements using patients indicated that physicians were the only ones who recommended the use of CAM to them.

Table 2 shows CAM use by cancer diagnosis type. The rate of patients using CAM varied between the individual diagnosis categories. While none of the ten leukemia patients took additional substances, a high rate of patients using CAM substances was found in myeloma patients. Eight out of 15 patients with multiple myeloma (53.3%) indicated using CAM supplements. Gynecologic cancer patients used CAM substances in 15 out of 40 cases (37.5%) what was about average. More women stated using CAM supplements (29 out of 68, 42.6%) than men (14 out of 47, 29.8%). The 29 women using CAM supplements took 83 different supplements (2.9 in average per patient, SD = 2.6), which included many different active ingredients whereas the 14 men using CAM supplements took 34 different CAM supplements in total (2.5 in average per patient, SD = 1.6).

The use of CAM supplements was statistically analyzed as shown in Table 3. Correlations were calculated for use (yes/no) and number of CAM supplements used with age (age over 61 years), gender, marital status, having children, having minor children, school leaving qualification, type of cancer diagnosis, time since initial cancer diagnosis (time longer than 1 year), and with the number of drugs prescribed (*n* > 9). Significant (*p* < 0.050) associations were found regarding the use of CAM supplements: Individuals older than 61 years were less likely to use CAM than younger patients (*p* = 0.020, φc = 0.229). When the time since diagnosis was longer than 1 year, the use of CAM supplements was significantly more likely (*p* = 0.006, φc = 0.264). A model of binary logistic regression including these two parameters to explain CAM use also showed significant results (Table 3), while the overall model fit was low (*χ*^2^ = 12.9). However, the number of different CAM compounds consumed did not correlate with any of these parameters.

**Table 3:** Statistical associations concerning CAM

Associations concerning the use of CAM supplements *			
With age over 61 years *	*n* = 115	*p* = 0.020, φc = 0.229 **	Significant, small effect size ***
With gender	*n* = 115	*p* = 0.176, φc = 0.131	No association
With marital status	*n* = 114	*p* = 0.223, φc = 0.220	No association
With having children *	*n* = 114	*p* = 0.369, φc = 0.103 **	No association
With having minor children *	*n* = 114	*p* = 0.073, φc = 0.176	No association
With school leaving qualification	*n* = 83	*p* = 0.845, φc = 0.131	No association
With type of cancer diagnosis	*n* = 115	*p* = 0.197, φc = 0.324	No association
With time since initial cancer diagnosis > 1 year *	*n* = 115	*p* = 0.006, φc = 0.264	Significant, small effect size ***
With number of drugs prescribed > 9 *	*n* = 115	*p* = 1.00, φc = 0.016 **	No association
Binary logistic regression model concerning the use of CAM supplements; *χ*^2^ = 12.9 *			
With age over 61 years *	*n* = 115	*p* = 0.035, OR = 0.422, CI95% [0.189, 0.939]	**Significant association**
With time since initial cancer diagnosis > 1 year *	*n* = 115	*p* = 0.011, OR = 3.042, CI95% [1.285, 7.196]	**Significant association**
Associations concerning the amount of CAM supplements used (*n*)			
With age over 61 years *	*n* = 115	*p* = 0.276, *η*^2^ = 0.010	No association
With gender	*n* = 115	*p* = 0.172, *η*^2^ = 0.016	no association
With marital status	*n* = 114	*p* = 0.391, *η*^2^ = 0.037	No association
With having children *	*n* = 114	*p* = 0.296, *η*^2^ = 0.010	No association
With having minor children *	*n* = 114	*p* = 0.102, *η*^2^ = 0.024	No association
With school leaving qualification	*n* = 83	*p* = 0.111, *η*^2^ = 0.073	No association
With type of cancer diagnosis	*n* = 115	*p* = 0.582, *η*^2^ = 0.076	No association
With time since initial cancer diagnosis > 1 year *	*n* = 115	*p* = 0.061, *η*^2^ = 0.031	No association
With number of drugs prescribed > 9 *	*n* = 115	*p* = 0.754, *η*^2^ = 0.001	No association
Binary logistic regression model concerning the potential of CAM-drug interactions with conventional cancer treatment; *χ*^2^ = 53.0 *			
With amount of CAM supplements used	*n* = 115	*p* < 0.001, OR = 3.660, CI95%[2.183,6.137]	**Significant association**
With amount of anticancer drugs taken	*n* = 115	*p* = 0.648, OR = 0.874, CI95%[.491,1.556]	No association

* Dichotomous variable: yes/no

** Negative directed associations regarding the use of CAM supplements

*** Small effect size: 0.100 < φc < 0.300 (Wei et al. 2019)

*n* refers to the number of patients whose data was considered for calculation

Potential CAM–drug interactions between CAM ingredients and conventional cancer treatment revealed in 22 of the 43 CAM supplements using patients (51.2%). There was a higher risk of CAM–drug interactions with anticancer drugs in patients taking a higher number of CAM compounds (*p* < 0.001, OR = 3.660, CI95% [2.183, 6.137]), but not when they received more drugs for cancer treatment (*p* = 0.648, OR = 0.874, CI95% [0.491, 1.556]). The overall model fit was *χ*^2^ = 53.0.

The potential risks of interactions with conventional cancer therapy were assessed based on literature data. For all active CAM ingredients taken by patients in our study, the potential interactions with conventional anticancer drugs are shown in Table 4. For comprehensiveness, regimes that were not prescribed to the patients of our study are also included. The actual occurrence in patients was not investigated in our study.

**Table 4:** Potential interactions between CAM and drugs used in conventional cancer therapy

Interactions with cancer treatment	
Vitamins	
Vitamin A	Possible: Hepatotoxic effects (García-Cortés et al. 2016). Caution when combining with drugs acting hepatotoxic such as cytarabine, daunorubicin, doxorubicin, epirubicin, gemcitabine, methotrexate, paclitaxel, topotecan, tretinoin, and others Possible: Reduction in the effects of anthracyclines and other regimes by antioxidative action (Zeller et al. 2013)
Vitamin B6	Possible: Reduced neurotoxicity of chemotherapy, but also reduction in its effectiveness. Study results based on hexamethylmelamine and cisplatin (Wiernik et al. 1992)
Vitamin B7	Unlikely
Vitamin B9 (Folate)	Possible: Neutropenia (Branda et al. 2004). Caution when combining with myelotoxic drugs such as multiple anticancer drugs Possible: Increase in effects of fluoropyrimidines such as fluorouracil and capecitabine, e.g., diarrhea (e.g., AbZ-Pharma GmbH 2016; HEUMANN PHARMA GmbH & Co. Generica KG 2015) Possible: Reduction in effects of methotrexate (e.g., AbZ-Pharma GmbH 2016; HEUMANN PHARMA GmbH & Co. Generica KG 2015)
Vitamin B12	Unlikely
Vitamin C	Likely: Reduction in effects of anthracyclines (Zeller et al. 2013) Likely: Reduction in effects of bortezomib (Perrone et al. 2009) Likely: Reduction in effects of bleomycin (Pohl and Reidy 1989) Likely: Reduction in effects of doxorubicin, cisplatin, vincristine, methotrexate, and imatinib (Heaney et al. 2008) Possible: Interactions with other regimes * (Zeller et al. 2013) * Due to the broad spectrum of interactions, especially due to antioxidative action, interactions with other chemotherapeutic agents than with the regimes investigated so far also seem possible Unlikely: Interactions with immunotherapy (Zeller et al. 2013)
Vitamin D	Unlikely
Vitamin E	Likely: Antagonistic effects of vitamin E and tamoxifen (Zeller et al. 2013) Possible: Reduction in effects of anthracyclines and other regimes by antioxidative action (Zeller et al. 2013) Possible: Reduction in effects of cisplatin and paclitaxel-based regimes while reduction in toxicity is reported for these regimes (Argyriou et al. 2006; Pace et al. 2003)
Vitamin K	Unlikely
Minerals	
Calcium	Likely: Additional Risk of hypercalcemia with tamoxifen (Arumugam et al. 2006)
Others	Unlikely (iron, magnesium, selenium, silicon, zinc)
Food and plant extracts	
Aloe vera	Possible: Carcinogen action (Guo and Mei 2016) Possible: Laxative effect can cause electrolyte imbalance and diarrhea. Hypokalemia is reported (Baretta et al. 2009; Guo and Mei 2016). Due to the laxative effect, a changed absorption of orally applied anticancer medication is also conceivable Unlikely: Plasma levels probably too low to achieve relevant potential inhibition of CYP2D6 or CYP3A4 (Djuv and Nilsen 2012)
Angocin	*Nasturtium and horseradish root* Unlikely
Beetroot	See: Calcium, vitamin C Possible: Reduction in effects of anthracyclines and other regimes by antioxidative action of betanin (Nestora et al. 2016)
Brazil nuts	Unlikely
Broccoli	Likely: Reduction in effects of cisplatin by GST-α induction (Allocati et al. 2018; Eagles et al. 2020) Possible: Reduction in effects of other regimes through GST modulation and e.g., resulting conferring of resistance to chemotherapy (Allocati et al. 2018) Possible: Reduction in effects of anthracyclines and other regimes by antioxidative action of sulphoraphane (Ferreira et al. 2018; Zeller et al. 2013)
Chinese herbs mixtures	Likely: Cytochrome (CYP) interactions (Zeller et al. 2013) depending on the different ingredients Likely: Interactions with endocrine therapy by phytoestrogens (Zeller et al. 2013) Possible: Other interactions (Zeller et al. 2013) depending on the different ingredients
Curcuma longa	Possible: Interactions by multiple cytochrome (CYP) effects and an inhibition of Pgp (Al-Jenoobi et al. 2015; Cho and Yoon 2015; Volak et al. 2008) Possible: Interactions with immunotherapy by multiple effects on the immune system and immunosuppressive action (Fahey et al. 2007; Kang et al. 1999; Skyvalidas et al. 2020) Possible: Reduced chemotherapy-induced apoptosis in cancer cells for camptothecin, cyclophosphamide, doxorubicin, mechlorethamine (Somasundaram et al. 2002), and other regimes by antioxidative effects * * Due to the broad spectrum of interactions, especially due to antioxidative action, interactions with other chemotherapeutic agents than with the regimes investigated so far also seem possible
Garlic	Possible: Interactions with bortezomib (CYP1A2), cisplatin (CYP2E1), and others by inhibition of CYP1A2, CYP2C9, and CYP2E1 (Cho and Yoon 2015; Foster et al. 2001; Ho et al. 2010; JANSSEN-CILAG INTERNATIONAL NV 2019; Lu and Cederbaum 2006; Quintanilha et al. 2017) Unlikely: Effects on Pgp, if existing, are rated as very low (Cho and Yoon 2015; Foster et al. 2001). No interactions regarding CYP2D6 or CYP3A4 (Cox et al. 2006; Markowitz et al. 2003)
Ginger	Likely: Interactions with bortezomib, cyclophosphamide, docetaxel, irinotecan, vincristine, and others by inhibition of CYP2C9, CYP2C19, and CYP3A4 (Cho and Yoon 2015; JANSSEN-CILAG INTERNATIONAL NV 2019; Kim et al. 2012; Kimura et al. 2010; Petri 2017; Qiu et al. 2015) Possible: Increase in effects of daunorubicin by inhibition of Pgp (Angelini et al. 2013; Nabekura et al. 2005)
Green tea extracts	Likely: Reduction in anticancer effects of boronic acid-based proteasome inhibitors like bortezomib by epigallocatechin gallate (EGCG) (Golden et al. 2009) Likely: Interactions with cyclophosphamide, docetaxel, irinotecan, tamoxifen, vincristine, and others by inhibition of Pgp and CYP3A4 (Chung et al. 2009; Engdal and Nilsen 2009; Petri 2017; Shin and Choi 2009). Variability in effect size by different extracts (Wanwimolruk et al. 2009) Possible: Hepatotoxic effects (García-Cortés et al. 2016; Mazzanti et al. 2009). Caution when combining with drugs acting hepatotoxic such as cytarabine, daunorubicin, doxorubicin, epirubicin, gemcitabine, methotrexate, paclitaxel, topotecan, tretinoin, and others
Hawthorn	Possible: Interactions with bortezomib, cyclophosphamide, docetaxel, irinotecan, vincristine, and others by induction of CYP3A4 (JANSSEN-CILAG INTERNATIONAL NV 2019; Petri 2017; Xu et al. 2011)
Lutein	Unlikely
Mistle	Likely: Increase in effects of paclitaxel by inhibiting ribosomal protein synthesis (Pae et al. 2001) Possible: Induction of hypersensitivity and interactions with immunotherapy by unspecific activation of the immune system (Zeller et al. 2013) Unlikely: Interactions regarding CYP3A4 (Engdal and Nilsen 2009; Schink and Dehus 2017)
Mushrooms (Medicinal mushrooms)	Possible: Cytochrome (CYP) interactions depending on the different ingredients. E.g., CYP2D6 induction by AHCC (Shitake mushrooms) leading in reduction in effects of doxurubicin (Mach et al. 2008) Possible: Induction of hypersensitivity and interactions with immunotherapy by unspecific activation of the immune system (Zeller et al. 2013)
Nigella sativa	Possible: Interactions with bortezomib, cyclophosphamide, docetaxel, irinotecan, vincristine, and others by inhibition of CYP2D6 and CYP3A4 (Al-Jenoobi et al. 2010; JANSSEN-CILAG INTERNATIONAL NV 2019; Petri 2017)
OPC (Oligomeric proantho-cyanidins)	Possible: Reduction in cytotoxic effects of cancer therapy. Investigated in a study for cyclophosphamide and idarubicin (Joshi et al. 2000). Heterogeneous data regarding doxurubicin (Li et al. 2010; Sharma et al. 2004)
Sage	Unlikely
Spirulina	Possible: Interactions with bortezomib (CYP1A2), bisplatin (CYP2E1), and others by inhibition of CYP1A2 and CYP2E1 (JANSSEN-CILAG INTERNATIONAL NV 2019; Lu and Cederbaum 2006; Quintanilha et al. 2017; Savranoglu and Tumer 2013)
Thistle (Milk thistle)	Unlikely: Plasma levels probably too low to achieve relevant potential inhibition of CYP3A4 or other CYP interactions or UGT modulation (Gurley et al. 2004; van Erp et al. 2005)
Thyme	Unlikely
Other processed CAM substances	
Coenzyme Q10 (Ubiquinone)	Possible: Interactions with multiple regimes * * Antioxidative acting Q10 concentrations are significantly increased in tumor cells (Portakal et al. 2000) accompanied by a reduced plasma concentration in patients with progressive cancer (Rusciani et al. 2006). It is controversial whether the additional intake of Q10 has a positive effect, or whether the reduction in the effects of various chemotherapies such as irinotecan, etoposid, doxorubicin, and methotrexate is the main focus (Huebner 2012 p86). A negative influence of the antioxidative enzyme has been shown for radiation therapy (Lund et al. 1998)
Detoxification infusion	See: Vitamins (mainly vitamin C), minerals, homeopathy
Homeopathy	Unlikely
Omega 3 fatty acids	Unlikely
Probiotics	Unlikely

## Discussion

37% of all patients reported using CAM supplements. This is in line with Loquai et al. (2016) who found that 34% used CAM supplements (biological-based CAM), and Alsanad et al. (2016) who reported a rate of 34% of all patients using herbal or dietary supplements. Molassiotis et al. (2005) found that 36% of all included European cancer patients used CAM of any modality with a range of 15% to 73% across countries, while CAM supplements such as herbal medicine or dietary supplements are the most frequently used CAM modalities in Europe and the United States (Alsanad et al. 2016; McCune et al. 2004; Micke et al. 2009; Molassiotis et al. 2005; Naing et al. 2011; Zeller et al. 2013). Data on the use of not only CAM supplements but all CAM modalities vary between regions, as reported even between European countries (Molassiotis et al. 2005). For Saudi Arabia, a percentage of 69.9% CAM use is reported among cancer patients mainly using religious CAM modalities and camel products (Abuelgasim et al. 2018).

Different demographic parameters of the patients are discussed in the literature as predictors of CAM use. The most commonly considered ones are female gender, younger age, higher education level, and breast cancer as the type of cancer diagnosis (Micke et al. 2009; Molassiotis et al. 2005; Naing et al. 2011; Richardson et al. 2000; Wode et al. 2019). Table 5 shows a selection of studies, which showed different results regarding the significance of various parameters within this context.

**Table 5:** Demographic parameters that may be suitable as predictors for CAM use according to different studies

Parameter	Considerable as a predictive parameter	
	Yes	No
Female gender	Micke et al. (2009) Molassiotis et al. (2005) Naing et al. (2011) Richardson et al. (2000) Wode et al. (2019)	**Our study**
Younger age	Micke et al. (2009) Molassiotis et al. (2005) Richardson et al. (2000) Wode et al. (2019) **Our study**	Naing et al. (2011)
Higher educational level	Micke et al. (2009) Molassiotis et al. (2005) Wode et al. (2019)	Naing et al. (2011) Richardson et al. (2000) **Our study**
Breast cancer	Micke et al. (2009)	Molassiotis et al. (2005) **Our study**
Longer time since initial cancer diagnosis	**Our study**	

Regarding younger age, our study also showed a significant association with CAM use. In addition, our results suggest that a longer period of time since initial diagnosis is also suitable as a predictor. Among the three most frequently mentioned reasons for using CAM in cancer patients are the attempt to reduce side effects of conventional therapy and the desire to become more active against the cancer disease (Huebner et al. 2014). This may explain why CAM use increases with the duration of disease: when side effects of conventional therapy first appear in the course of the disease, or when patients’ desire to influence the course of their cancer themselves increases as the disease progresses. Although the number of patients using CAM supplements in our study differed within the diagnosis categories, no significant association was found between type of cancer diagnosis and CAM use. A higher rate (64–76%) of CAM users among breast cancer patients described by some authors (Huebner et al. 2014; Zeller et al. 2013) could not be confirmed. In fact, breast cancer patients used CAM slightly less frequently than the average of all patients (36% vs. 37%). Random influences might affect our results, as the total number of breast cancer patients was small (*n* = 25) compared to other authors (Micke et al. 2009; Molassiotis et al. 2005). Nevertheless, even with these authors, the data are inconclusive. The international multicenter study by Molassiotis et al. identified cancer entities others than breast cancer with higher rates of CAM using patients (2005). In contrast to other authors, we could not determine gender as a reliable predictor of CAM use (Micke et al. 2009; Molassiotis et al. 2005; Naing et al. 2011; Richardson et al. 2000; Wode et al. 2019). As described by Richardson et al. (2000) too, we could not detect a significant association with marital status. Having one or more children or minor children as well as the consumption of a high number of conventional drugs did not prove to be suitable predictors either.

Yet, the use of individual parameters as predictors of CAM usage should be treated with caution. Calculated effect sizes, when indicated, were small and the models could only explain a small part between 6 and 26% of the difference between the groups of CAM users and non-CAM users (Molassiotis et al. 2005; Richardson et al. 2000; Wode et al. 2019), as was the case also in our study. In all patients, regardless of age, gender, cancer diagnosis, etc., CAM use should be investigated and documented by physicians in charge to identify potential interactions with conventional cancer therapy.

More than half (51%) of all patients using CAM supplements were at risk of CAM–drug interactions involving their cancer treatment prescribed by physicians. This is less than has been reported by other authors who used a similar classification system on potential interactions as we did. Up to 65% (Zeller et al. 2013) and even 85% (Loquai et al. 2017) are reported elsewhere. A lower number of interactions compared with the studies mentioned above might occur if the patients in our study took fewer CAM supplements per person than in the other study. Other authors focused on a particular source of CAM–drug interactions, such as interactions via CYP enzymes and Pgp (P-glycoprotein) interactions (Engdal et al. 2009) and examined lower interaction rates, as might be expected. In addition, authors of different studies evaluate the likelihood of CAM–drug interactions differently, so results based on different classifications (e.g., Engdal et al. 2009; Lee et al. 2006; McCune et al. 2004; Werneke et al. 2004) appear difficult to compare.

Our analyses showed that the potential of CAM–drug interactions with cancer therapy was significantly related only to the number of CAM supplements taken but, to our surprise, not to the number of anticancer medication prescribed by physicians. This could be explained partly by the fact that CAM substances have a rather dichotomous pattern of potential interactions, according to our research: in many cases, CAM substances either had a very wide range of potential interactions, such as curcuma longa or vitamin C, or interactions were generally unlikely, as is the case with most minerals. Therefore, the number of CAM products taken may be critical because as the number of CAM substances increases, the likelihood increases that there will be a CAM supplement among them that has a broad spectrum of interactions. A smaller number of CAM products taken is more likely to be products for which no potential interactions have been explored. Conventional drugs, on the other hand, seem to have a more evenly distributed interaction spectrum, so how many are taken is less relevant to the overall potential for interactions (yes/no).

For some CAM substances, bioavailability is high and substantial serum concentrations of the active ingredients can be reached easily, which increases the risk of interactions. Taking vitamin C as an example, several studies have shown reduced effects of chemotherapy regarding a variety of therapeutic regimes: anthracyclines, bortezomib, bleomycin, cisplatin, vincristine, methotrexate, and imatinib (Heaney et al. 2008; Perrone et al. 2009; Pohl and Reidy 1989; Zeller et al. 2013). In addition, reduced toxicity of various chemotherapeutic drugs with the use of antioxidants is discussed, such as for oligomeric proanthocyanidins (OPC) with regard to anthracyclines or cyclophosphamide (Joshi et al. 2000; Li et al. 2010; Sharma et al. 2004), while the influence on the efficacy of cytostatic therapy has not yet been sufficiently studied (Huebner 2012 p342). A recent large cohort study by Jung et al. (2019) showed that taking antioxidant supplements during radio- or chemotherapy is associated with higher mortality as well as a reduced recurrence-free survival.

Interactions are difficult to denote in individual patients, as their influence on the course of the disease cannot be proven in most cases. To protect patients from such interactions, proactive recommendations not to use supplements which might entail interactions seem appropriate. Therefore, physicians should strive to know about supplement use of the patients they care for. Only about one-third (33–36%) of all patients consuming CAM supplements talked to their physician about it (Firkins et al. 2018; Kennedy 2005). Consideration and documentation of CAM supplements by treating physicians seems important and necessary to identify possible interactions. This requires knowledge of the benefits, risks, and potential interactions of CAM supplements by physicians and pharmacists. A study published in 2009 investigating drug interactions with CAM supplements complained of a lack of literature on potential interactions in 48% of the herbal remedies used by patients (Engdal et al. 2009). Although many more studies have been published since then, most of the data still bases on mouse models or in vitro experiments (e.g., Huebner 2012). Clinical trials, data from large cohorts and registries, and more efforts to obtain reliable information are essential to effectively counsel patients based on human data.

In a U.S. study, more than 50% of CAM using patients indicated that CAM supplements were important for their well-being and health (Kennedy 2005). On the other hand, the patients do not want to harm themselves. Education about the possible consequences of interactions is important. Moreover, patients’ desire to be active, participate in treatment, and reduce side effects while improving quality of life should be acknowledged, and physicians should offer advice on safe CAM methods, which may be certain supplements but also healthy lifestyle, nutrition, and physical activity.

### Limitations

An important limitation is the lack of data and the presence of few to none studies on the interaction potential regarding many CAM agents. Despite careful consideration, the assessments of the probability of CAM interactions are partly vague because of insufficient literature or inconsistent assessments by other authors. Other authors might reach different conclusions for certain CAM supplements. The specific numbers calculated might not be sufficient for generalization since the study was carried out at one center with cancer patients with different cancer diagnoses. Random influences might affect the results on associations, especially regarding the highest school-leaving qualification due to a smaller number of included cases and the diagnosis of breast cancer due to a small number of breast cancer patients overall (25 out of 115 patients). The severity and frequency of any clinical manifestation of the interactions was not studied but should be investigated in further studies. The potential interactions identified in this study provide the basis for this.

## Conclusion

Interactions of conventional cancer therapy with over-the-counter CAM supplements are often underestimated and yet insufficiently researched. High doses of supplements in the form of extracts and concentrates should be avoided, especially in cancer patients, if there is no proven medical indication for their use, e.g., a deficiency of a micronutrient. Overdoses should be avoided in all cases. Physicians should address risks and document the use of CAM supplements in patient records and check for interactions. Further studies are needed for a variety of CAM supplements regarding benefits and risks, such as interaction risks.

## Data Availability

The datasets generated and analyzed during the current study are available from the corresponding author on reasonable request.
